# The safety and tolerability of combined immune checkpoint inhibitors (anti-PD-1/PD-L1 plus anti-CTLA-4): a systematic review and meta-analysis

**DOI:** 10.1186/s12885-019-5785-z

**Published:** 2019-06-10

**Authors:** Lihu Gu, Parikshit Asutosh Khadaroo, Hui Su, Liya Kong, Liangliang Chen, Xianfa Wang, Xinlong Li, Hepan Zhu, Xin Zhong, Junhai Pan, Manman Chen

**Affiliations:** 1 Department of General Surgery, HwaMei Hospital, University of Chinese Academy of Sciences, Ningbo, Zhejiang China; 2 Monash University School of Public Health and Preventive Medicine, Melbourne, Australia; 3 Zhejiang Chinese Medical University, Hangzhou, Zhejiang China; 4 Department of Surgical Oncology, HwaMei Hospital, University of Chinese Academy of Sciences, Ningbo, Zhejiang China; 5 Department of General Surgery, Zhejiang University School of Medicine Sir Run Run Shaw Hospital, Hangzhou, Zhejiang China; 6 Affiliated Hospital of Medical School Ningbo University and Ningbo City Third Hospital, No. 247, Renming Road, Ningbo, 315020 Zhejiang China

**Keywords:** PD-1, PD-L1, CTLA-4, Adverse events, Meta-analysis

## Abstract

**Background:**

The future of combined immunotherapy (a PD-1/PD-L1 plus a CTLA-4 antagonist) is very bright. However, besides improving efficacy, combined therapy increases treatment-related adverse events (TRAEs). Also, the clinical application is limited in some solid tumors.

**Methods:**

This paper purports to investigate the TRAEs for the combined immunotherapy aiming for a more appropriate utilization of immune checkpoint inhibitors (ICIs) in clinical practice through a meta-analysis.

**Results:**

A total of 17 eligible studies covering 2626 patients were selected for a meta-analysis based on specified inclusion and exclusion criteria. The incidence rates of any grade and grade 3 or higher TRAEs were 88% (95%CI, 84–92%) and 41% (95%CI, 35–47%), respectively. The overall incidence of any grade TRAEs leading to discontinuation of treatment was 20% (95%CI, 16–24%). The incidence rate of treatment related deaths was 4.3‰ (95%CI, 1.4‰-8.4‰). Analysis showed that NIVO1 + IPI3 cohort had higher incidences of grade 3 or higher TRAEs (RR = 1.77, 95%CI, 1.34–2.34, *p* < 0.0001) and any grade TRAEs leading to discontinuation of treatment (RR = 1.81, 95%CI, 1.08–3.04, *P* = 0.02), compared with NIVO3 + IPI1 regimen.

**Conclusions:**

The combined therapy had high TRAEs. The TRAEs, especially grade 3 or higher, led to discontinuation of the treatment. Furthermore, the incidence of treatment-related deaths was rare. Moreover, the NIVO3 + IPI1 regimen, regardless of efficacy, is more recommended because of better tolerance and lower adverse events.

**Electronic supplementary material:**

The online version of this article (10.1186/s12885-019-5785-z) contains supplementary material, which is available to authorized users.

## Background

Immunotherapies that involve immune checkpoint inhibitors (ICIs) comprise cytotoxic T lymphocyte-associated protein-4 (CTLA-4), programmed cell death protein-1 and ligand-1 (PD-1 and PD-L1) monoclonal antibodies. The first ICI, ipilimumab, received the US Food and Drug Administration (FDA) approval in 2011 [1]. It was then approved for treatment of advanced melanoma. Since 2014, PD-1/PD-L1 inhibitors, especially nivolumab, became an established treatment option for a number of tumors, including melanoma [2], non-small cell lung cancer (NSCLC) [3], renal cell carcinoma (RCC) [4] and Hodgkin’s lymphoma [5]. Furthermore, the combined use of one CTLA-4 plus one PD-1/PD-L1 antagonist was rather more efficacious than the respective monotherapies in some solid tumors [6, 7].

Combination or sole use of ICI potentiates some form of toxicity profiles which were never observed previously [8, 9]. They are known as immune-related adverse events (irAEs) which include thyroid dysfunction, colitis, pneumonitis, dermatitis as well as hepatitis amongst others [8, 9]. The aetiology of these toxicities are autoimmune and are peculiarly different from toxicities observed with conventional cytotoxic chemotherapy. ICIs usually causes side effects associated with autoimmune diseases by altering immune-surveillance [9, 10]. There are a few meta-analyses on the incidences of irAEs to date, and most include articles observed monotherapy or one ICI combined with chemotherapy [11, 12]. In brief, although the combined immunotherapy (anti-PD-1/PD-L1 plus anti-CTLA-4) improves the efficacy, there is a prominent increase of side effects. Consequently, the clinical use of combined ICIs is challenged.

Although most of the combined immunotherapy has achieved encouraging results in an increasing number of published reports for various tumors [13, 14], no one has ever conducted a meta-analysis of the treatment-related adverse events (TRAEs) for anti-PD-1/PD-L1 plus anti-CTLA-4. As a matter of fact, the combined immunotherapy involves different treatment regimens. We conducted a systematic review and meta-analysis of trials of anti-PD-1/PD-L1 plus anti-CTLA-4 in patients with tumor and compared the incidence of TRAEs among the groups treated with different combination regimens. We believe this meta-analysis will help enhance awareness of the incidence and characteristics of TRAEs, which may lead to a more appropriate utilization of ICIs in clinical practice for combination therapy.

## Methods

### Search methods

The following databases were systematically searched for literatures about clinical trials of combined ICIs (anti-PD-1/PD-L1 and anti-CTLA-4): PubMed, EBSCO, Web of Science and Cochrane Library. The databases were searched for articles published on or before September 2018. The search terms used were: (“nivolumab”OR “BMS 936558” OR “BMS 936559” OR “MDX 1105” OR “pembrolizumab” OR “lambrolizumab” OR “MK 3475” OR “pidilizumab” OR “CT 011” OR “durvalumab” OR “MEDI 4736” OR “atezolizumab” OR “MPDL 3280a” OR “avelumab” OR “AMP 224”) AND (“ipilimumab” OR “tremelimumab”). Additionally, the reference lists of the selected articles were individually reviewed to obtain other potentially relevant articles. Original articles published with prospective clinical trials of the combined ICIs for patients with advanced solid tumors were selected, including adverse events. Selected publications were all in English language.

### Study selection

For inclusion in this meta-analysis: (1) investigated the safety and efficacy of the combined ICIs for treatment of solid tumors; (2) clearly reported the adverse events in their safety data, with or without clinical severity grading. The exclusion criteria are listed as follows:(1) the combined ICIs regimens included other therapies, such as chemotherapy and targeted therapy; (2) two immune checkpoint blockade were not used concurrently; (3) the original articles were presented only as meeting abstracts without published full-text; (4) the trials also covered non-solid tumors, such as lymphoma. In the event of duplicates, ambiguity, or publications reporting on the same study population, only the most recent, relevant, and/or comprehensive publication was included in the analysis.

### Data extraction

Data from each included study was extracted by two investigators and reviewed independently by a third investigator following the Preferred Reporting Items for Systematic Review and Meta-Analysis guidelines [15]. Any discrepancy in study selection was resolved by consensus. The number of patients treated with combined ICIs, the number of patients with any grade TRAEs, the number of patients with grade 3 or higher TRAEs, the number of patients with TRAEs leading to treatment discontinuation, the number of patients with grade 3 or higher TRAEs leading to treatment discontinuation, the number of patients with any grade treatment-related serious adverse events, the number of patients with each TRAEs, and the number of treatment-related deaths were extracted. The trial phases, tumor types, types of specific agents, dose, and frequency of drug administration were recorded. The incidence of TRAEs was characterized based on all grades and grade 3 or higher as reported by each trial using the definitions of National Cancer Institute’s Common Terminology Criteria for Adverse Events (CTCAE).

### Statistical analysis

For each clinical trial, the number of patients treated and the number of patients with adverse events reported were recorded for each treatment arm and dose level. All models were fit using log/logit/arcsine/Freeman-Tukey Double arcsine transformation, respectively, and restricted maximum likelihood estimation using an offset of 0.5 for all 0 cells. According to the normal test results, select the appropriate transformation. Heterogeneity was evaluated using the Cochran Q statistic and I^2^ statistics for its proportion of the total variability. If *p* ≥ 0.1 and I^2^ ≤ 50%, there is homogeneity between the results of the study, and the fixed-effect model will be used for meta-analysis; if *p* < 0.1 and I^2^ < 50%, heterogeneity exists among the results within an acceptable range, and the fixed-effect model is still required; if p < 0.1 and I^2^ ≥ 50%, it indicates that there is substantial heterogeneity among the data, and that we should analyze the source of the heterogeneity in an alternative way. Only when there is no apparent clinical heterogeneity, can the random-effect model be used for meta-analysis cautiously. Publication bias was evaluated by Egger’s test, p < 0.1 was considered statistically significant. Egger’s test of publication bias was not performed on analysis subgroup with less than 10 studies because of low sensitivity of qualitative and quantitative tests. All analyses were performed in Revman 5.3 Software and R 3.4.3 (meta and metafor package).

## Results

### Eligible studies and characteristics

The search strategy originally generated 4342 relevant clinical trials from the databases. After screening and eligibility assessment, a total of 17 eligible studies [7, 13, 14, 16–29] were selected for this meta-analysis, including 2626 patients. The detailed search and study selection process is shown in Additional file 1: Figure S1. Tumor types tested in these studies included melanoma (*n* = 6), NSCLC (*n* = 3), RCC (*n* = 2), small cell lung cancer (*n* = 1), colorectal cancer (n = 1), glioblastoma (n = 1), esophagogastric cancer (n = 1), mesothelioma (n = 1) and sarcoma (n = 1) (Additional file 3: Table S1). Almost patients had an Eastern Cooperative Oncology Group (ECOG) performance status of 0 or 1 in 14 studies, except for six patients. Patients had a Karnofsky performance status (KPS) score of at least 70% (in two studies) or at least 80% (in one study).

### Therapeutic regimens of combination therapy

The regimens were categorized by class as: nivolumab plus ipilimumab cohort (*n* = 14) and other combination cohort (n = 3). The latter including pembrolizumab plus ipilimumab (n = 1) and durvalumab plus tremelimumab (n = 2). According to dose, and frequency of drug administration, the nivolumab plus ipilimumab cohort was further divided into three subgroups: NIVO1 + IPI3 cohort, NIVO3 + IPI1 cohort and other NIVO+IPI cohort. The regimens of NIVO1 + IPI3 cohort (*n* = 8) was nivolumab 1 mg/kg plus ipilimumab 3 mg/kg, every 3 weeks for 4 doses (induction phase), followed by nivolumab 3 mg/kg, every 2 weeks until disease progression or unacceptable toxicity incidence of TRAEs (maintenance phase). The regimens of NIVO3 + IPI1 cohort (*n* = 7) was nivolumab 3 mg/kg plus ipilimumab 1 mg/kg, every 3 weeks for 4 doses (induction phase), followed by nivolumab 3 mg/kg, every 2 weeks until disease progression or unacceptable toxicity incidence of TRAEs (maintenance phase). The regimens of other NIVO+IPI cohort (*n* = 4) were variable, as shown in Additional file 3: Table S1.

### Incidence of TRAEs

**Fig. 1:**
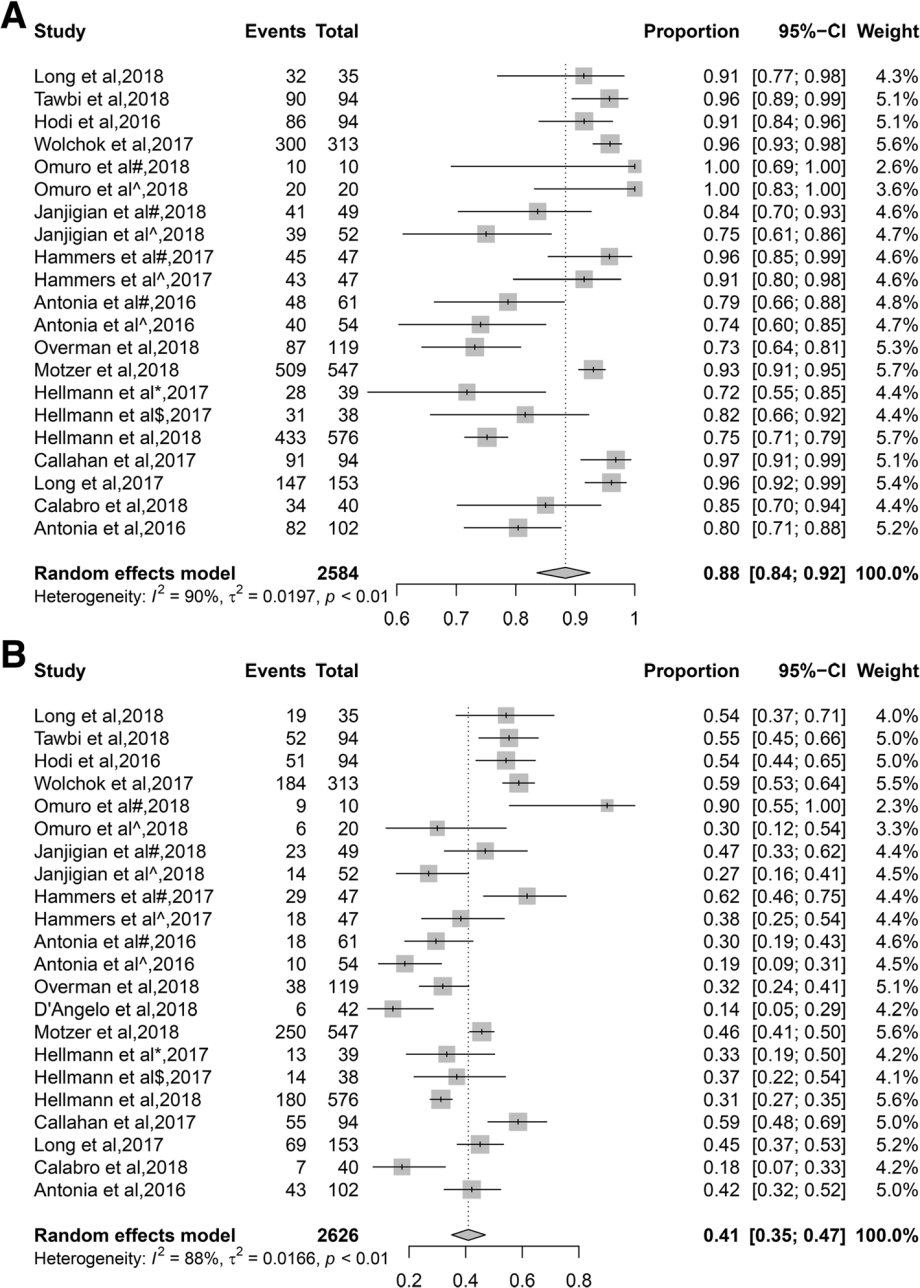
Forest plots of the incidence of TRAEs for combined immunotherapy (anti-PD-1/PD-L1 and anti-CTLA-4). **a** any grade TRAEs, **b** grade 3 or higher TRAEs

16 articles reported any grade TRAEs, of which the incidence ranged from 72 to 100%, and the incidence rate was 88% (95%CI, 84–92%). The incidence was 95 and 76% in melanoma and NSCLC patients, respectively. The incidence was 92 and 86% in NIVO1 + IPI3 cohort and NIVO3 + IPI1 cohort, respectively. 17 articles reported grade 3 or higher TRAEs, the incidence ranged from 14 to 90%, and the overall incidence rate was 41% (95%CI, 35–47%). The incidence was 55 and 33% in melanoma and NSCLC patients, respectively. The incidence was 54 and 29% in NIVO1 + IPI3 cohort and NIVO3 + IPI1 cohort, respectively (Fig. 1 and Table 1).

**Table 1:** Subgroup analysis and characteristics of the TRAEs

	No. of studies	No. of patients	Incidence	95%CI	Effects model	Heterogeneity		Egger’s test (p)
						(I^2^)	p	
Any grade TRAEs	16	2584	0.88	0.84–0.92	Random	90%	< 0.01	0.97
Any grade TRAEs (melanoma)	6	783	0.95	0.93–0.96	Fixed	0%	0.43	
Any grade TRAEs (NSCLC)	3	755	0.76	0.73–0.79	Fixed	0%	0.51	
Any grade TRAEs (NIVO1 + IPI3)	8	703	0.92	0.88–0.96	Random	70%	< 0.01	
Any grade TRAEs (NIVO3 + IPI1)	6	839	0.86	0.75–0.95	Random	90%	< 0.01	
Any grade TRAEs(Phase II-III trials)	8	1818	0.89	0.81–0.95	Random	95%	< 0.01	
Grade 3 or higher TRAEs	17	2626	0.41	0.35–0.47	Random	88%	< 0.01	0.83
Grade 3 or higher TRAEs (melanoma)	6	783	0.55	0.51–0.58	Fixed	40%	0.14	
Grade 3 or higher TRAEs (NSCLC)	3	755	0.33	0.30–0.36	Fixed	38%	0.019	
Grade 3 or higher TRAEs (NIVO1 + IPI3)	8	703	0.54	0.46–0.62	Random	75%	< 0.01	
Grade 3 or higher TRAEs (NIVO3 + IPI1)	7	881	0.29	0.20–0.40	Random	86%	< 0.01	
Grade 3 or higher TRAEs (Phase II-III trials)	9	1860	0.40	0.31–0.50	Random	93%	< 0.01	
Any grade TRAEs leading to discontinuation of treatment	17	2626	0.20	0.16–0.24	Random	82%	< 0.01	0.09
Any grade TRAEs leading to discontinuation of treatment (melanoma)	6	783	0.30	0.20–0.39	Random	89%	< 0.01	
Any grade TRAEs leading to discontinuation of treatment (NSCLC)	3	755	0.18	0.13–0.27	Random	70%	0.02	
Any grade TRAEs leading to discontinuation of treatment(NIVO1 + IPI3)	8	703	0.27	0.19–0.35	Random	81%	< 0.01	
Any grade TRAEs leading to discontinuation of treatment(NIVO3 + IPI1)	7	881	0.14	0.10–0.20	Random	63%	0.01	
Any grade TRAEs leading to discontinuation of treatment (Phase II-III trials)	9	1860	0.22	0.16–0.29	Random	90%	< 0.01	
Grade 3 or higher TRAEs leading to discontinuation of treatment	10	1538	0.16	0.12–0.23	Random	83%	< 0.01	0.28
Grade 3 or higher TRAEs leading to discontinuation of treatment(melanoma)	4	595	0.28	0.24–0.31	Fixed	36%	0.20	
Grade 3 or higher TRAEs leading to discontinuation of treatment(NSCLC)	2	653	0.12	0.09–0.14	Fixed	0%	0.38	
Any grade TRAEs leading to discontinuation of treatment (Phase II-III trials)	6	1236	0.17	0.11–0.27	Random	91%	< 0.01	
Any grade treatment-related serious adverse events	10	1270	0.32	0.27–0.39	Random	71%	< 0.01	0.28
Treatment-related deaths	17	2626	0.0043	0.0014 −0.0084	Fixed	0%	0.77	0.95
Any grade fatigue	15	2484	0.38	0.30–0.46	Random	92%	< 0.01	0.40
Any grade diarrhea	15	2551	0.29	0.24–0.35	Random	87%	< 0.01	0.25
Any grade pruritus	14	2450	0.26	0.20–0.31	Random	90%	< 0.01	0.26
Any grade rash	16	2586	0.22	0.17–0.29	Random	92%	< 0.01	0.04
Any grade nausea	13	2348	0.20	0.16–0.25	Random	82%	< 0.01	> 0.99
Any grade hypothyroidism	12	2331	0.14	0.13–0.16	Fixed	40%	0.06	0.86
Any grade decreased appetite	11	2135	0.14	0.11–0.17	Random	52%	< 0.01	0.15
Grade 3 or higher increased lipase	13	1790	0.09	0.06–0.12	Random	80%	< 0.01	0.43
Grade 3 or higher colitis	10	1166	0.06	0.04–0.08	Random	50%	0.02	0.65
Grade 3 or higher increased ALT	13	1428	0.06	0.04–0.09	Random	72%	< 0.01	0.93
Grade 3 or higher increased AST	13	1428	0.05	0.02–0.07	Random	72%	< 0.01	0.75
Grade 3 or higher diarrhea	15	2551	0.05	0.03–0.07	Random	79%	< 0.01	0.38
Grade 3 or higher fatigue	15	2484	0.02	0.01–0.03	Random	58%	< 0.01	0.52
Grade 3 or higher rash	16	2586	0.01	0.01–0.02	Fixed	24%	0.15	0.79

*TRAEs* Treatment-related adverse events, *NSCLC* Non-small cell lung cancer, *NIVO1 + IPI3* Nivolumab 1 mg/kg plus ipilimumab 3 mg/kg, every 3 weeks for 4 doses (induction phase), followed by nivolumab 3 mg/kg, every 2 weeks until disease progression or unacceptable toxicity incidence of TRAEs (maintenance phase); NIVO3 + IPI1, nivolumab 3 mg/kg plus ipilimumab 1 mg/kg, every 3 weeks for 4 doses (induction phase), followed by nivolumab 3 mg/kg, every 2 weeks until disease progression or unacceptable toxicity incidence of TRAEs (maintenance phase)

### Incidence of TRAEs leading to discontinuation of treatment and serious adverse events

**Fig. 2:**
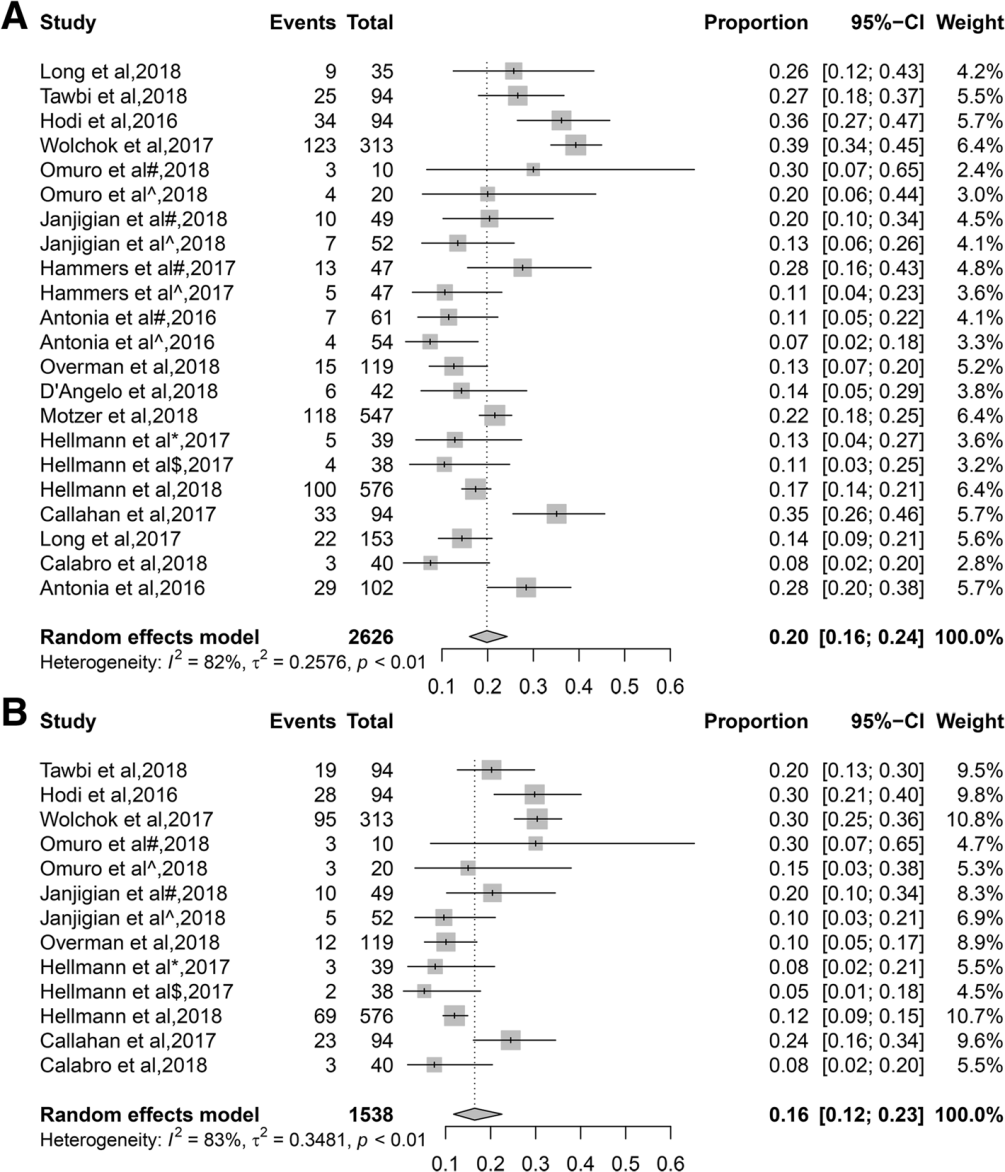
Forest plot of the incidence of TRAEs leading to discontinuation of treatment for combined immunotherapy (anti-PD-1/PD-L1 and anti-CTLA-4). **a** any grade TRAEs leading to discontinuation of treatment, **b** grade 3 or higher TRAEs leading to discontinuation of treatment

Any grade TRAEs leading to discontinuation of treatment was reported by 17 articles, the incidence ranged from 7 to 39%, and the overall incidence was 20% (95%CI, 16–24%). The incidence was 30 and 18% in melanoma and NSCLC patients, respectively. The incidence was 27 and 14% in NIVO1 + IPI3 cohort and NIVO3 + IPI1 cohort, respectively. 10 articles reported grade 3 or higher TRAEs leading to discontinuation of treatment, the incidence ranged from 5 to 30%, and the overall incidence rate was 16% (95%CI, 12–23%). The incidence was 28 and 12% in melanoma and NSCLC patients, respectively. Besides, 10 of the articles reported any grade treatment-related serious adverse events, the incidence of which ranged from 23 to 70%, and the overall incidence rate was 32% (95%CI, 27–39%) (Fig. 2 and Table 1).

### Incidence of treatment-related deaths

All included articles reported treatment-related deaths, and the incidence rate was 4.3‰ (95%CI, 1.4‰-8.4‰). A total of 29 deaths were related to study drugs. The most common causes were pulmonary events (*n* = 9) and cardiac events (*n* = 7). Pneumonitis was the most frequent cause of death in respiratory adverse drug reaction. Cardiac events included myocarditis, ventricular arrhythmia and cardiac tamponade. Other cause of deaths included hepatic necrosis, renal failure and myasthenia gravis. In addition, there were also some rare causes including hemo-phagocytic syndrome and tumor lysis syndrome (Additional file 2: Figure S2).

### Incidence of common TRAEs

The most common any grade TRAEs were fatigue (38%), diarrhea (29%), pruritus (26%), rash (22%), and nausea (20%). The most common grade 3 or higher TRAEs were increased lipase (9%), colitis (6%), increased ALT (6%), increased AST (5%), and diarrhea (5%) (Table 1).

### NIVO1 + IPI3 vs. NIVO3 + IPI1 regimens

**Fig. 3:**
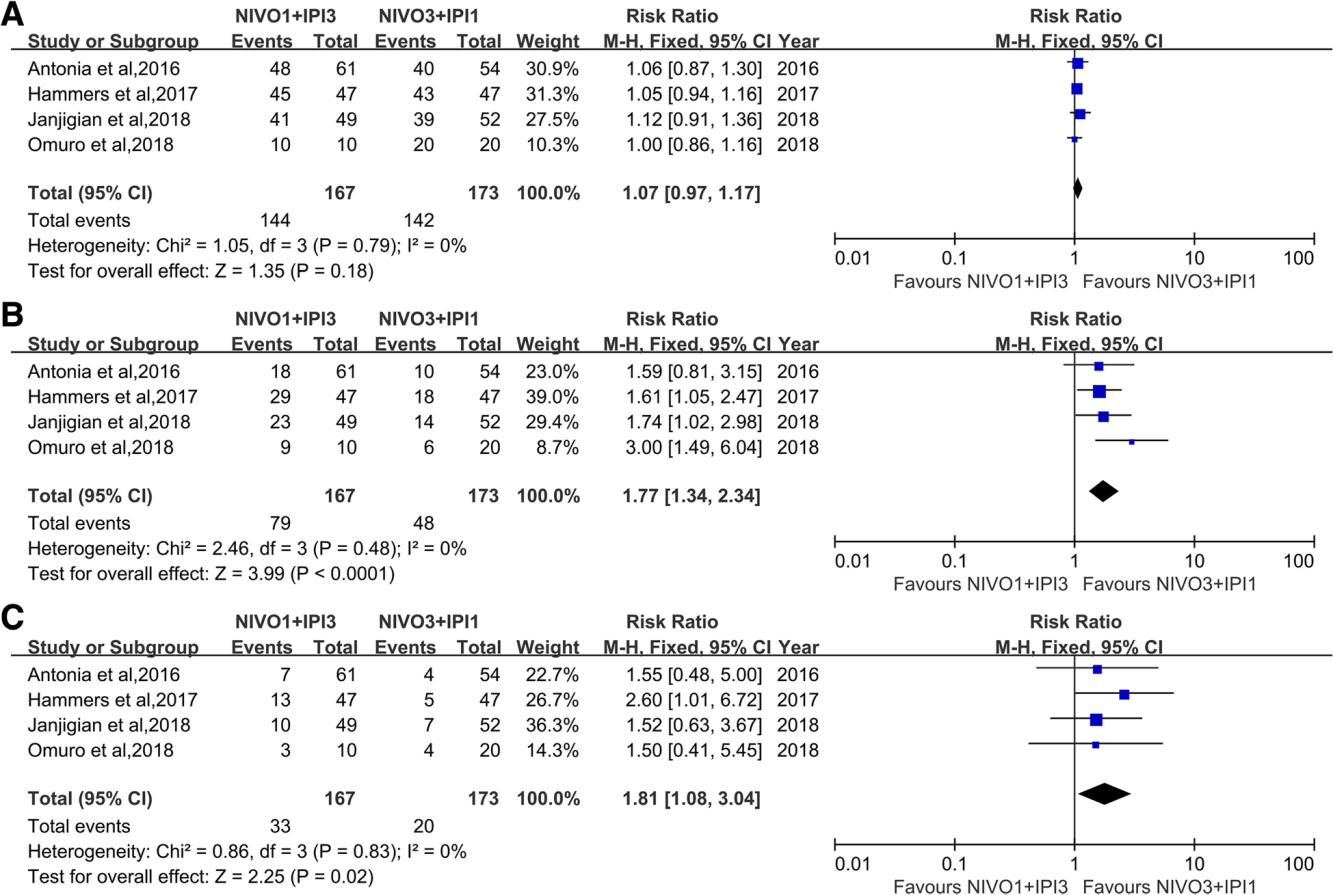
Forest plot describing the association between the tolerability and therapeutic regimens (NIVO1 + IPI3 vs NIVO3 + IPI1). **a** any grade TRAEs, **b** grade 3 or higher TRAEs, **c** any grade TRAEs leading to discontinuation of treatment

4 studies investigated and compared the activity and safety of nivolumab combined with ipilimumab (NIVO1 + IPI3 vs. NIVO3 + IPI1). Analysis showed that NIVO1 + IPI3 cohort had more grade 3 or higher TRAEs (RR = 1.77, 95%CI, 1.34–2.34, *p* < 0.0001). Meanwhile, the any grade TRAEs leading to discontinuation of treatment was more likely to occur in patients with NIVO1 + IPI3 regimen too (RR = 1.81, 95%CI, 1.08–3.04, *p* = 0.02). Although not statistically significant, a slightly higher likelihood of any grade TRAEs was noted in patients with NIVO1 + IPI3 compared with NIVO3 + IPI1 cohort (RR = 1.07, 95%CI, 0.97–1.17, *p* = 0.18) (Fig. 3).

## Discussion

To the best of our knowledge, this is the first meta-analysis to investigate the adverse drug events for combined ICIs (anti-PD-1/PD-L1 plus anti-CTLA-4). To date, most clinical trials of combination immunotherapy have chosen a treatment regimen of nivolumab combined with ipilimumab, namely NIVO1 + IPI3 or NIVO3 + IPI1 regimen [30]. One-third of the patients recruited for the clinical trial were with advanced melanoma in this article.

This study demonstrated the incidence of TRAEs among patients who had received combination therapy. Most patients had at least one any grade TRAEs during treatment course. Additionally, about half of the patients had higher grade TRAEs. Most importantly, a considerable number of patients discontinued the treatment because of TRAEs. In a word, patients with serious adverse events were not in the minority and TRAEs needed a more serious consideration.

With a frequency of up to 38%, fatigue was the most common adverse event. In contrast to the frequent occurrence, their severity was normally low (2% Grade 3 or higher). The findings were consistent with report by Sznol et al. [31]. Similarly, nearly one-third of cases had diarrhea, but most were not in poor condition except for those with colitis. Other common adverse events, such as pruritus, rash, nausea and so on, were relatively mild.

Increased lipase, with an overall incidence of approximately one-tenth, was the most common grade 3 or higher TRAEs. D’Angelo et al. reported that the most common treatment-related grade 3 or 4 adverse events were lipase elevation and diarrhea in combination therapy [32]. Su et al. summarized relevant clinical trials and found that combination treatment can significantly increase the risk of grade 3 or higher lipase elevation as well as any grade amylase elevation, compared with nivolumab or ipilimumab alone. However, neither monotherapy nor combination therapy are seen to potentiate the risk of immune-induced pancreatitis. The precise and specific mechanism for such observed differences are still unknown [33]. Consistent with previous results of Wang et al., about 6% patients had grade 3 or higher colitis [34]. Sznol et al. comprehensively analyzed a randomized clinical trial published recently and their results showed a higher rate of TRAEs leading to discontinuation of combination therapy than those with monotherapy of nivolumab or ipilimumab. The most common cause of discontinuation was colitis and diarrhea in all studies [31]. They were observed to be the primary immune-related gastrointestinal events and would have the same adverse drug reaction. Schadendorf et al. came to the conclusion that colitis was the most frequently reported TRAEs, which led to discontinuation rate of 10% [35]. Hepatotoxicity was also one of the important irAEs in immunotherapy. The current research showed that elevation of ALT and AST were only second to lipase elevation and colitis in grade 3 or higher TRAEs. Wang et al. reported that CTLA-4 inhibitors are linked to a higher risk of hepatotoxicity compared to PD-1 inhibitors [36].

In this study, the most common treatment-related deaths were caused by pneumonitis and cardiac causes. Baxi et al [37] found that the pneumonitis was the most common serious irAEs in monotherapy (PD-1/PD-L1 antagonist). However, our study shows that pneumonia was not the most common serious TRAEs but the most important cause of treatment-related deaths. Therefore, clinicians should pay great attention to pneumonia, especially of grade 3 or higher in immunotherapy. On the other hand, previous research had suggested that cardiac effects of ICIs were highly variable, but myocarditis was the most reported form of ICI-associated cardiotoxicity, as this cardiotoxicity is generally reversible with corticosteroids [38].

Generally, combination therapy had high incidence of TRAEs, including any grade TRAEs, grade 3 or higher TRAEs, and any grade treatment-related serious adverse events. In addition, any grade TRAEs, mainly about grade 3 or higher TRAEs, leading to discontinuation occurred in one in five patients. Thus, TRAEs, grade 3 or higher TRAEs in particular, became one of the major problems that could not be ignored in combination therapy. Meanwhile, it was also one of the most important factors limiting clinical application and reducing effects. However, the incidence of treatment-related deaths was low (< 1%), because the majority of events was reversible after the systemic use of glucocorticoids, then, well and safely managed. As described by Hassel et al., “in case of long-lasting and/or refractory immune-toxicities, organ- or case-specific escalation of immunosuppression was recommended” [30].

Notably, through subgroup analysis, it found that although any grade TRAEs of NIVO1 + IPI3 treatment regimen were similar to these of NIVO3 + IPI1, the former was associated with a higher incidence of grade 3 or higher TRAEs compared with the latter. Analogously, the analysis reinforced the fact that patients treated with NIVO1 + IPI3 had a higher risk of interruption due to any grade TRAEs than those treated with NIVO3 + IPI1. This is the first meta-analysis to report that NIVO3 + IPI1 regimen has a better tolerance than NIVO1 + IPI3 for clinical trials of combined ICIs (anti-PD-1/PD-L1 plus anti-CTLA-4).

Interestingly, Fujii et al. [39, 40] investigated the relationship between irAEs and response to the treatment. The results showed that irAEs had been associated with improved treatment outcomes, suggestive of an active immune status. So, the side effects of treatment needed to be reevaluated. Furthermore, the complexity of tumor microenvironment and also the intertwined tumor and immune cells interaction, it is however very hard to develop strong biomarkers that could help identify patients who may respond to immunotherapy [41].

### Limitations

The limitations of this study should be stressed on. The heterogeneity among included studies cannot be ignored. Despite subgroup analysis, the heterogeneity persists. One of the important reasons is that the patient characteristics are quite different, such as race, geographic region, ECOG, tumor type, PD-L1 expression level and so on. Medication dose might be another important reason. More importantly, the combined immunotherapy regimens are mainly nivolumab plus ipilimumab, and therefore conclusions of this study cannot be applied to other ICIs.

## Conclusions

This meta-analysis first evaluated TRAEs incidence of the combined therapy (anti-PD-1/PD-L1 plus anti-CTLA-4), and the regimen has a high incidence of TRAEs. Also, the TRAEs, especially grade 3 or higher, lead to treatment discontinuation. Pulmonary and cardiac toxicity were the leading causes of treatment-related death, but the incidence of treatment-related deaths was low. Last but not least, regardless of efficacy, the NIVO3 + IPI1 regimen is recommended as a combination treatment due to better tolerance and lower adverse events.

## Additional files

Additional file 1: **Figure S1.** Flow diagram of study inclusion and exclusion. (DOCX 78 kb)

Additional file 2: **Figure S2.** Forest plot of the incidence of treatment-related to deaths for combined immunotherapy (anti-PD-1/PD-L1 and anti-CTLA-4). (TIF 1446 kb)

Additional file 3: **Table S1.** Characteristics of included trials in the meta-analysis. (DOCX 19 kb)

## Data Availability

The datasets supporting the conclusions of this article are included within the article.

## Abbreviations

ALT: Alanine aminotransferase
AST: Aspartate aminotransferase
CI: Confidence interval
CTCAE: Common Terminology Criteria for Adverse Events
CTLA-4: Cytotoxic T lymphocyte-associated protein-4
ECOG: Eastern Cooperative Oncology Group
FDA: Food and Drug Administration
ICIs: Immune checkpoint inhibitors
irAEs: Immune-related adverse events
KPS: Karnofsky performance status
NSCLC: Non-small cell lung cancer
PD-1 and PD-L1: Programmed cell death protein-1 and ligand-1
RCC: Renal cell carcinoma
RR: Relative risk
TRAEs: Treatment-related adverse events
