# Supplementary material for: The safety and tolerability of combined immune checkpoint inhibitors (anti-PD-1/PD-L1 plus anti-CTLA-4): a systematic review and meta-analysis
Source: BMC Cancer. 2019 Jun 10;19:559. doi: 10.1186/s12885-019-5785-z (PMC6558837; doi:10.1186/s12885-019-5785-z)
Supplement: Supplementary file 1 — Figure S1. Flow diagram of study inclusion and exclusion. (DOCX 78 kb) [file 12885_2019_5785_MOESM1_ESM.docx]

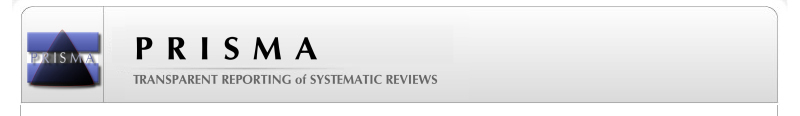
**PRISMA 2009 Flow Diagram**

Full-text articles excluded, with reasons
(n =50)

Non-prospective clinical trials (n=22)

Sequential therapy with combined immune checkpoint inhibitors (n=15)

The combined regimens included other therapies (n= 9)

Previous studies with updated cohort data (n=4)

Additional file 1: **Figure S1** Flow diagram of study inclusion and exclusion

Studies included in quantitative synthesis (meta-analysis)
(n =17)

Studies included in qualitative synthesis
(n =17)

Full-text articles assessed for eligibility
(n =67)

Records excluded
(n =1307)

Records screened
(n =1374)

Records after duplicates removed
(n =1374)

Additional records identified through other sources
(n =5)

Identification

Eligibility

Included

Screening

Records identified through database searching
(n = 4337)
