# Supplementary figures and images for: The safety and tolerability of combined immune checkpoint inhibitors (anti-PD-1/PD-L1 plus anti-CTLA-4): a systematic review and meta-analysis
Source: BMC Cancer. 2019 Jun 10;19:559. doi: 10.1186/s12885-019-5785-z (PMC6558837; doi:10.1186/s12885-019-5785-z)

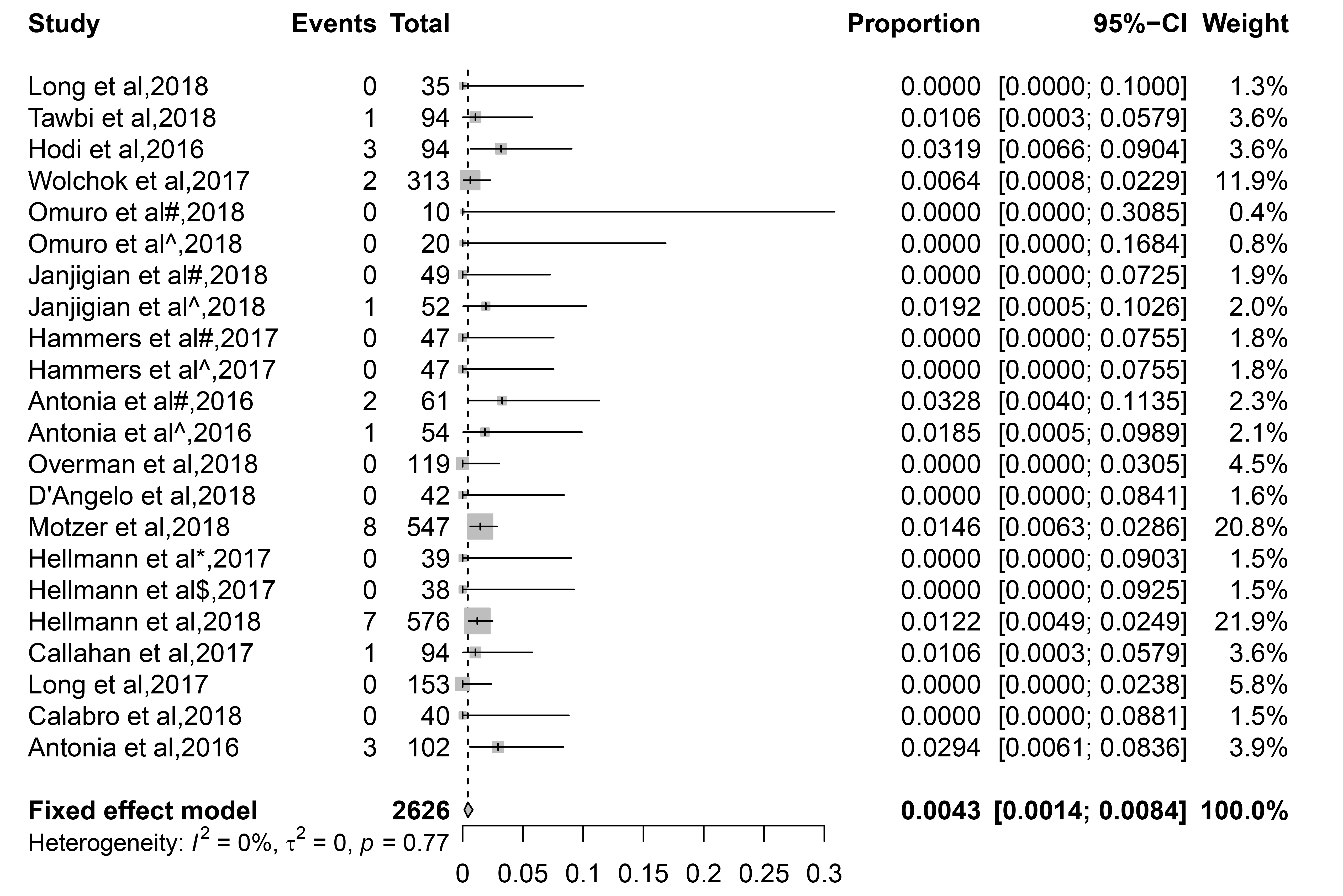

Supplement: Supplementary file 2 — Figure S2. Forest plot of the incidence of treatment-related to deaths for combined immunotherapy (anti-PD-1/PD-L1 and anti-CTLA-4). (TIF 1446 kb) [file 12885_2019_5785_MOESM2_ESM.tif]
