# Supplementary material for: The safety and tolerability of combined immune checkpoint inhibitors (anti-PD-1/PD-L1 plus anti-CTLA-4): a systematic review and meta-analysis
Source: BMC Cancer. 2019 Jun 10;19:559. doi: 10.1186/s12885-019-5785-z (PMC6558837; doi:10.1186/s12885-019-5785-z)
Supplement: Supplementary file 3 — Table S1. Characteristics of included trials in the meta-analysis. (DOCX 19 kb) [file 12885_2019_5785_MOESM3_ESM.docx]

**Additional file 3:** Table S1 **Characteristics of included trials in the meta-analysis**

| Author,  year | Trials | Clinicaltrials.gov,  number | Phase | Tumor type | NO. of patients | Treatment comparsion (anti-PD-1/PD-L1+anti-CTLA-4 antibodies) |
| --- | --- | --- | --- | --- | --- | --- |
| Long et al,  2018 | NA | NCT02374242 | 2 | Melanoma | 35 | nivolumab(1mg/kg)+ipilimumab(3mg/kg) Q3W 4 doses(induction phase);  nivolumab(3mg/kg) Q2W(maintenance phase) |
| Tawbi et al,  2018 | Checkmate 204 | NCT02320058 | 2 | Melanoma | 94 | nivolumab(1mg/kg)+ipilimumab(3mg/kg) Q3W 4 doses(induction phase);  nivolumab(3mg/kg) Q2W(maintenance phase) |
| Hodi et al,  2016 | Checkmate 069 | NCT01927419 | 2 | Melanoma | 94 | nivolumab(1mg/kg)+ipilimumab(3mg/kg) Q3W 4 doses(induction phase);  nivolumab(3mg/kg) Q2W(maintenance phase) |
| Wolchok et al,  2017 | Checkmate 067 | NCT01844505 | 3 | Melanoma | 313 | nivolumab(1mg/kg)+ipilimumab(3mg/kg) Q3W 4 doses(induction phase);  nivolumab(3mg/kg) Q2W(maintenance phase) |
| Omuro et al^#^,  2018 | Checkmate 143 | NCT02017717 | 1 | Glioblastoma | 10 | nivolumab(1mg/kg)+ipilimumab(3mg/kg) Q3W 4 doses(induction phase);  nivolumab(3mg/kg) Q2W(maintenance phase) |
| Omuro et al^^^,  2018 | Checkmate 143 | NCT02017717 | 1 | Glioblastoma | 20 | nivolumab(3mg/kg)+ipilimumab(1mg/kg) Q3W 4 doses(induction phase);  nivolumab(3mg/kg) Q2W(maintenance phase) |
| Janjigian et al^#^,  2018 | Checkmate 032 | NCT01928394 | 1/2 | Esophagogastric  cancer | 49 | nivolumab(1mg/kg)+ipilimumab(3mg/kg) Q3W 4 doses(induction phase);  nivolumab(3mg/kg) Q2W(maintenance phase) |
| Janjigian et al^^^,  2018 | Checkmate 032 | NCT01928394 | 1/2 | Esophagogastric  cancer | 52 | nivolumab(3mg/kg)+ipilimumab(1mg/kg) Q3W 4 doses(induction phase);  nivolumab(3mg/kg) Q2W(maintenance phase) |
| Hammers et al^#^,  2017 | Checkmate 016 | NCT01472081 | 1 | RCC | 47 | nivolumab(1mg/kg)+ipilimumab(3mg/kg) Q3W 4 doses(induction phase);  nivolumab(3mg/kg) Q2W(maintenance phase) |
| Hammers et al^^^,  2017 | Checkmate 016 | NCT01472081 | 1 | RCC | 47 | nivolumab(3mg/kg)+ipilimumab(1mg/kg) Q3W 4 doses(induction phase);  nivolumab(3mg/kg) Q2W(maintenance phase) |
| Antonia et al^#^,  2016 | Checkmate 032 | NCT01928394 | 1/2 | SCLC | 61 | nivolumab(1mg/kg)+ipilimumab(3mg/kg) Q3W 4 doses(induction phase);  nivolumab(3mg/kg) Q2W(maintenance phase) |
| Antonia et al^^^,  2016 | Checkmate 032 | NCT01928394 | 1/2 | SCLC | 54 | nivolumab(3mg/kg)+ipilimumab(1mg/kg) Q3W 4 doses(induction phase);  nivolumab(3mg/kg) Q2W(maintenance phase) |
| Overman et al,  2018 | Checkmate 142 | NCT02060188 | 2 | CRC | 119 | nivolumab(3mg/kg)+ipilimumab(1mg/kg) Q3W 4 doses(induction phase);  nivolumab(3mg/kg) Q2W(maintenance phase) |
| D'Angelo et al,  2018 | A091401 | NCT02500797 | 2 | Sarcoma | 42 | nivolumab(3mg/kg)+ipilimumab(1mg/kg) Q3W 4 doses(induction phase);  nivolumab(3mg/kg) Q2W(maintenance phase) |
| Motzer et al,  2018 | Checkmate 214 | NCT02231749 | 3 | RCC | 547 | nivolumab(3mg/kg)+ipilimumab(1mg/kg) Q3W 4 doses(induction phase);  nivolumab(3mg/kg) Q2W(maintenance phase) |
| Hellmann et al^*^,  2017 | Checkmate 012 | NCT01454102 | 1 | NSCLC | 39 | nivolumab(3mg/kg) Q2W+ipilimumab(1mg/kg) Q6W |
| Hellmann et al^$^,  2017 | Checkmate 012 | NCT01454102 | 1 | NSCLC | 38 | nivolumab(3mg/kg) Q2W+ipilimumab(1mg/kg) Q12W |
| Hellmann et al,  2018 | Checkmate 227 | NCT02477826 | 3 | NSCLC | 576 | nivolumab(3mg/kg) Q2W+ipilimumab(1mg/kg) Q6W |
| Callahan et al,  2017 | CA209-004 | NCT01024231 | 1 | Melanoma | 94 | nivolumab(1mg/kg)+ipilimumab(3mg/kg) Q3W 4 doses(induction phase);  nivolumab(3mg/kg) Q2W(maintenance phase) (41 patients) |
| Long et al,  2017 | Keynote 029 | NCT02089685 | 1 | Melanoma | 153 | pembrolizumab(2mg/kg)+ipilimumab(1mg/kg) Q3W 4 doses(induction phase);  pembrolizumab(2mg/kg) Q3W(maintenance phase) |
| Calabro et al,  2018 | NIBIT-MESO-1 | NCT02588131 | 2 | Mesothelioma | 40 | durvalumab(20mg/kg)+tremelimumab(1mg/kg) Q4W 4 doses(induction phase);  durvalumab(20mg/kg) Q4W 9 doses(maintenance phase) |
| Antonia et al,  2016 | NA | NCT02000947 | 1 | NSCLC | 102 | durvalumab +tremelimumab |

NA, not available; RCC, renal cell carcinoma; SCLC, small cell lung cancer; CRC, colorectal cancer; NSCLC, non-small cell lung cancer, ^#^ and ^^^ belong to same study, ^*^ and ^$^ belong to same study; ^#^ nivolumab(1mg/kg)+ipilimumab(3mg/kg) Q3W 4 doses(induction phase); ^^^ nivolumab(3mg/kg)+ipilimumab(1mg/kg) Q3W 4 doses(induction phase); ^*^ nivolumab(3mg/kg) Q2W+ipilimumab(1mg/kg) Q6W; ^$^ nivolumab(3mg/kg) Q2W+ipilimumab(1mg/kg) Q12W
